# Identification of Two Wnt-Responsive Elements in the Intron of RING Finger Protein 43 (RNF43) Gene

**DOI:** 10.1371/journal.pone.0086582

**Published:** 2014-01-22

**Authors:** Norihiko Takahashi, Kiyoshi Yamaguchi, Tsuneo Ikenoue, Tomoaki Fujii, Yoichi Furukawa

**Affiliations:** Division of Clinical Genome Research, Advanced Clinical Research Center, Institute of Medical Science, The University of Tokyo, Tokyo, Japan; University of Texas Medical Branch, United States of America

## Abstract

RING finger protein 43 (RNF43), an E3-type ubiquitin ligase, is frequently up-regulated in human colorectal cancer. It has been shown that expression of *RNF43* is regulated by the Wnt-signaling pathway. However the regulatory region(s) for its transcriptional activation has not been clarified. In this study, we have shown for the first time that *RNF43* is a direct target of TCF4/β-catenin complex, and that its expression is regulated by a regulatory region containing two Wnt-responsive elements (WREs) in intron2. A reporter gene assay revealed that nucleotide substitutions in the WREs decreased the reporter activity in colon cancer cells, suggesting that both WREs are involved in the transcriptional activation. Knockdown of β-catenin by siRNA suppressed the reporter activity. In addition, ChIP assay showed that both elements associate with TCF4/β-catenin complex in colon cancer cells. These data indicate that expression of *RNF43* is regulated by the canonical Wnt/β-catenin pathway through binding of the WREs with TCF4/β-catenin complex. These findings should be useful for the understanding of the regulatory mechanism of RNF43 and may contribute to the clarification of signaling pathways regulated by RNF43.

## Introduction

Colorectal cancer is one of the most common malignancies worldwide, and the third most common cancer-related death in Japan and in the United States of America. In the US, it is estimated that 142,820 of new cases will be diagnosed and that 50,830 patients will die of this disease in 2013 (SEER stat fact sheets, http://seer.cancer.gov/statfacts/html/colorect.html) [1]. Although tumors at early stages are cured by surgery, those with far advanced stages are not curable by operation alone. Molecular targeted drugs, such as bevacizumab, cetuximab, and panitumumab, have been approved for the combination therapies to advanced colorectal cancer, and have improved the efficacy of chemotherapies. Nevertheless, the five-year survival rate of metastatic cancer is still lower than 12% [1], suggesting that novel therapeutic strategies are needed.

Molecular studies have clarified that deregulation of the Wnt signaling pathway is involved in colorectal carcinogenesis. Wnt signal regulates differentiation, proliferation, compartmentation, and cell fate of epithelial cells in the intestinal mucosa. One of the key mediators of the pathway is β-catenin, which also plays a structural role in cell-cell adhesion by binding to cadherins [2]. In the absence of Wnt signaling, a multi-molecular complex comprising of β-catenin, APC, Axin/Axin2 (or Conductin) and glycogen synthase kinase 3β (GSK3β) phosphorylates β-catenin, leading to its ubiquitination and subsequent degradation in the proteosome [3]. Upon binding with the Frizzled family and LRP receptor complexes, Wnt proteins activate Dishevelled (Dvl) proteins that inhibit activity of glycogen synthase kinase 3β [4]. As a result, degradation of β-catenin is suppressed and accumulated β-catenin induces TCF/LEF-mediated transcription [5], [6].

In colorectal cancer cells, frequent mutations are observed in *APC*, the responsible gene for familial adenomatous polyposis of the colon, and *β-catenin (CTNNB1)* [4], [7]. In hepatocellular carcinomas, frequent mutations are found in *CTNNB1* and *AXIN1* [4], [8], [9]. These mutations are mutually exclusive, and result in transactivation of TCF/LEFs, members of high mobility group (HMG) box protein family [5], [9], suggesting that mutation in one of these components is enough to abrogate canonical Wnt signaling, and that TCF/LEF mediated transcriptional activation is important for these tumors. It has been thought that TCF4/β-catenin complex bend the DNA to access distant DNA region and form correct chromatin conformations for efficient RNA polymerase II (pol II)-mediated transcription [10]. Consequently, downstream target genes such as *c-myc* [11], *cyclinD1* [12], *MMP-7* (*matrilysin*) [13], [14], *urokinase-type plasminogen activator receptor* (*uPAR*) [15], *connexin 43* [16], *CD44* [17], *PPAR-delta* [18], *AF17* [19], *ENC1* [20], *Laminin-5 γ2* [21], *Claudin-1* [22] and *MT1-MMP* [23] are activated.

Earlier assigned as a hypothetical protein FLJ20315, RNF43 was shown to be an ubiquitin E3 ligase that associates with a nuclear protein, HAP95 [24]. Recently, two groups revealed that RNF43 enhances degradation of Wnt receptors including frizzled. One group showed that the degradation is mediated by the interaction with R-spondin proteins [25], and the other reported that this is carried out by endocytosis in LGR5-positive stem cells in the intestine [26]. Interestingly, *RNF43* mutations were identified in a subset of pancreatic cancer [27], [28], cholangiocarcinoma [29], colorectal cancer [30], and mucinous ovarian cancer [31]. These findings suggested that RNF43 is an important regulator of Wnt/β-catenin as well as Wnt/PCP pathway. In our earlier study, we found that expression of *RNF43* was frequently enhanced in colorectal cancer as well as hepatocellular carcinomas [32], [33]. In addition, other groups revealed that *RNF43* expression was also elevated in adenomas of the colon [34], that it is down-regulated by a dominant-negative form of Tcf4 in LS174 colon cancer cells [35], and that expression of *RNF43* was induced by Wnt3a conditioned media [25]. These data suggested that *RNF43* is a downstream gene regulated by the Wnt-signaling pathway, but none has clarified the regulatory regions of its expression. In this study, we identified two Wnt-responsive elements (WREs) in intron2 of *RNF43* and found that these WREs are crucial for its transcriptional regulation through interaction with Tcf4/β-catenin complex. This is the first report of *RNF43* as a direct target of Tcf4/β-catenin complex and our data may be useful to understand the precise mechanism of RNF43 regulation.

## Materials and Methods

### Cell Lines

Human colorectal cancer cell lines, HCT116 and SW480, were obtained from the American Type Culture Collection (Manassas, VA). HCT116 cells were cultured in McCoy’s 5A medium containing 10% fetal bovine serum (FBS, Life Technologies, Carlsbad, CA) and antibiotic/antimycotic solution (Sigma, St. Louis, MO). SW480 cells were cultured in Leibovitz’s L-15 medium containing 10% FBS and antibiotic/antimycotic solution.

### Gene Silencing

Human *CTNNB1*-specific siRNA were purchased from Dharmacon (ON-TARGETplus SMARTpool siRNA, L-003482-00). ON-TARGETplus Non-targeting Pool (D-001810-10) was used as a control. HCT116 or SW480 were seeded a day before treatment of siRNA. Cells were transfected with 15 nM of *CTNNB1*-specific or control siRNA using Lipofectamine RNAiMAX (Life Technologies). After 48 hours incubation, total RNAs were isolated with miRNeasy Mini Kit (Qiagen, Valencia, CA) according to the manufacture’s instruction. The silencing effect was evaluated by quantitative RT-PCR and western blotting. Complementally DNA was synthesized from 1 µg of total RNA with Transcriptor First Strand cDNA Synthesis Kit (Roche Diagnostics GmbH, Mannheim Germany). Real-time PCR was performed using SYBR Green technology with sets of primers (*RNF43*: forward primer, 5′-GTTTGCTGGTGTTGCTGAAA-3′, reverse primer, 5′-TGGCATTGCACAGGTACAG-3′, *GAPDH*: forward primer, 5′-AGCCACATCGCTCAGACA-3′, reverse primer, 5′-GCCCAATACGACCAAATCC-3′) for *RNF43* on StepOnePlus (Life Technologies). Amounts of transcripts were determined by relative standard curve method, and *GAPDH* was used as internal control.

### Preparation of Reporter Plasmids

Putative promoter regions in the 5′-flanking region of RNF43 were amplified by PCR with two sets of primers (forward primer, 5′-AAAACGCGTCTACAGGGGAAACAATGTTGAAGGTCAATAGGCT-3′, and reverse primer, 5′-AAACTCGAGTGGCCAGGTTTCTAGGCCCACTGC-3′ or 5′-AAACTCGAGTGGCAAAGAGAATGCCAACTGGTGCTGT-3′, containing a recognition site of *Mlu*I or *Xho*I restriction enzyme (underlined nucleotides). PCR products were digested with the restriction enzymes and cloned into the appropriate enzyme sites of pGL3-Basic vector (Promega, Madison, WI). In addition, putative intronic enhancer region was amplified by PCR with sets of primers (fragment 1+2; 5′-AAAACGCGTAGACTATTTGGCTGTCTCAAAGTCATTGCC-3′ and 5′-AAACTCGAGCCAGGGCCCAGCATTGTGCCT-3′, fragment 1; 5′-AAAACGCGTAGACTATTTGGCTGTCTCAAAGTCATTGCC-3′ and 5′-AAACTCGAGTGGGGCATAGGCCCTGGTG-3′, fragment 2; 5′-AAAACGCGTCACCAGGGCCTATGCCCCAC-3′ and 5′-AAACTCGAGCCAGGGCCCAGCATTGTGCCT-3′, containing a recognition site of *Mlu*I or *Xho*I restriction enzyme (underlined nucleotides). PCR products were digested with the restriction enzymes, and cloned into the appropriate enzyme sites of pGL3-Promoter vector (Promega). Site-directed mutagenesis was carried out for both putative TCF4 binding sites, replacing CTTTGWW by CTTTGGC with the QuickChange II XL Site-Directed Mutagenesis Kit (Agilent Technologies, Santa Clara, CA) according to the manufacture’s instruction.

### Luciferase Assay

HCT116 or SW480 cells seeded on six-well plates were transfected with 1 µg of reporter plasmid and 0.1 µg of pRL-TK plasmid (Promega) by FuGENE 6 reagent (Roche) and incubated for 12 hours. Then the cells were further transfected with *CTNNB1* siRNA or control siRNA (ON-TARGETplus Non-targeting Pool #D-001810-10) at the concentration of 15 nM and incubated for an additional 36 hours. The cells were harvested and luciferase activities were measured using dual luciferase assay system (TOYO B-Net, Tokyo, Japan).

### Chromatin Immunoprecipitation (ChIP) Assay

A ChIP assay was performed according to the Agilent Mammalian ChIP protocol with slight modifications. HCT116 or SW480 cells were cross-linked with 1% formaldehyde for 10 minutes at room temperature and quenched with 0.4 M glycine. Chromatin extracts were sheared by Micrococcal nuclease digestion, and subsequently protein-DNA complexes were immunoprecipitated with 3 µg of anti-TCF4 monoclonal antibody (6H5-3, Upstate, Charlottesville, VA) or anti-β-catenin monoclonal antibody (14/β-catenin, BD Transduction Laboratories, Franklin Lakes, NJ) bound to anti-mouse IgG-coated Dynabeads (Life Technologies). Non-immune mouse IgG (Santa Cruz Biotechnology, Santa Cruz, CA) was used as a negative control. The precipitated protein-DNA complexes were purified with the conventional DNA extraction method, and the DNAs were subjected to quantitative PCR analysis with the following primer sets; RNF43-int2-5′, forward primer, 5′-TCAACTCTCTGGATAAGGTGGAATAGC-3′, and reverse primer, 5′-GACTTTTGGGGTGGGTGGGAAATA-3′; RNF43-int2-3′, forward primer, 5′- TCGGGCACCTGGCCAAGATACA-3′, and reverse primer, 5′- TGGACGCCCTGGCTTCTGAG-3′. Specificity of the assay was determined by the amplification of a 5′-flanking region located from −4861 to −4768 of *RNF43* transcriptional start site using the following primers; forward primer, (−4861) 5′- CAAGGCTAGTCTGCCTCCAG-3′, reverse primer, (−4768) 5′- AGCGCTTTCCAAAGGAGGAA-3′. In addition, the amplifications of c-Myc (*MYC*) enhancer was used as a positive control (forward primer, 5′- GCTCAGTCTTTGCCCCTTTGTGG-3′, reverse, 5′- AACACCTTCCCGATTCCCAAGTG-3′).

## Results

### Knockdown of β-catenin Suppresses *RNF43*

**Figure 1:**
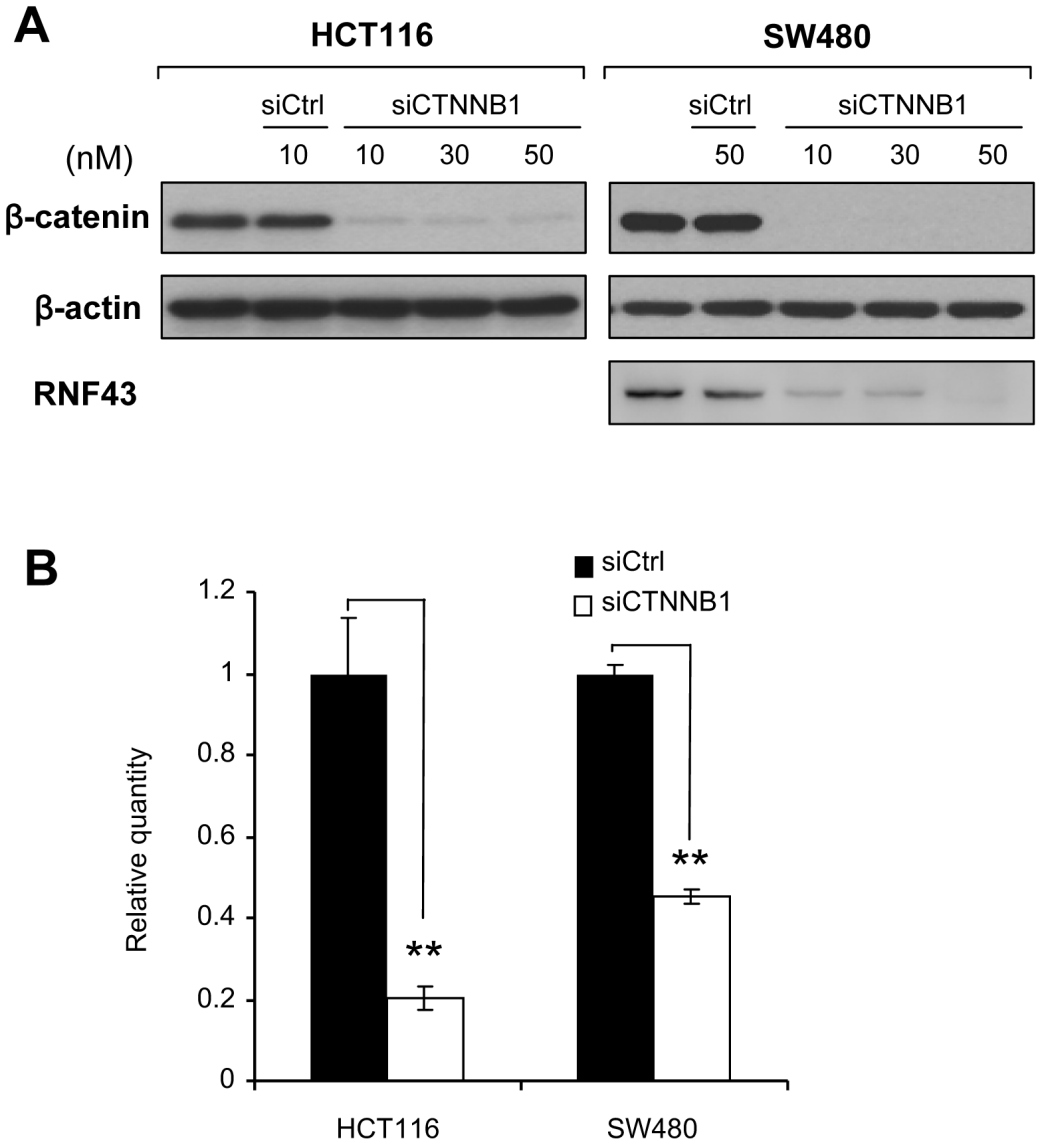
Effect of β-catenin depletion on *RNF43* expression. A) Knock down of β-catenin using *CTNNB1*-specific siRNA (siCTNNB1). HCT116 and SW480 cells were treated with siCTNNB1 or siCtrl, at the concentrations indicated in the figure. Expression levels of β-catenin and RNF43 were detected by western blotting with β-catenin- and RNF43-specific antibodies, respectively. B) Quantitative RT-PCR was carried out in triplicate using RNA from the cells. Cells were treated with 15 nM of *CTNNB1* siRNA (siCTNNB1) or control siRNA (siCtrl) for 48 hours. Relative expression of *RNF43* to the control siRNA is shown (mean ± standard deviation). A significant difference was determined by Student’s t-test. **; P<0.01.

To confirm that *RNF43* is regulated by the Wnt/β-catenin pathway, we measured expression levels of *RNF43* with or without β-catenin siRNA in HCT116 and SW480 cells (Figure 1A). HCT116 and SW480 cells exhibited constitutive activation of Wnt/β-catenin pathway through a mutation in *CTNNB1* or *APC*, respectively. Expectedly, quantitative RT-PCR disclosed that *RNF43* transcripts were markedly decreased by the depletion of β-catenin in these cells (Figure 1B). Consistently the protein level of RNF43 was reduced in SW480 cells treated with siCTNNB1 (Figure 1A). RNF43 protein was not detected in HCT116 cells because they harbor a homozygous mutation of *RNF43*. Since *RNF43* transcripts were more markedly decreased by siCTNNB1 in HCT116 than SW480, we used HCT116 cells for the analysis of regulatory region(s).

### Promoter Analysis of *RNF43*

**Figure 2:**
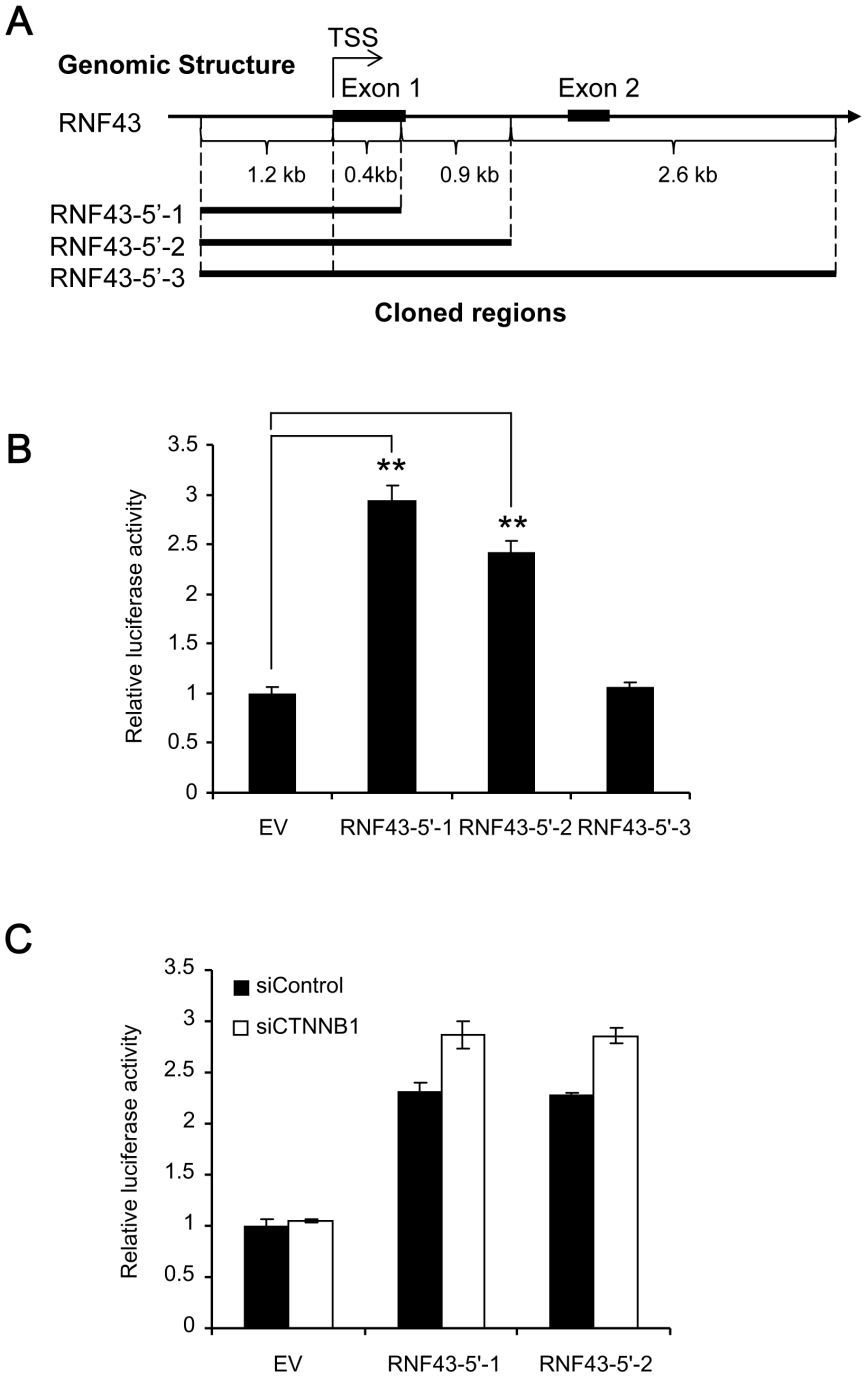
Promoter activity of the TSS-flanking region of *RNF43.* A) A genomic map of the TSS-flanking region of RNF43, and schematic representation of inserted regions in the reporter plasmids (pGL3-basic). TSS: transcription start site. B) Promoter activities of the reporter plasmids (mean ± standard deviation, **; P<0.01, Student’s t-test). C) Effect of β-catenin depletion on the promoter activities (RNF43-5′-1 and RNF43-5′-2). EV: empty vector.

To identify the regulatory element(s) of Wnt-signaling in *RNF43*, we constructed three forms of reporter plasmids (RNF43-5′-1, RNF43-5′-2 and RNF43-5′-3) containing the 5′-flanking region and intronic regions of *RNF43*. RNF43-5′-1 contained approximately 1.6 kb (chr17∶56494505–56496131, GRCh37), RNF43-5′-2 approximately 2.5 kb (chr17∶56493599–56496131, GRCh37) and RNF43-5′-3 approximately 5.1 kb (chr17∶56491044–56496131, GRCh37) of *RNF43*, respectively (Fig. 2A). These regions contained two elements, 5′-CTTTGAG-3′and 5′-CTTTGTC-3′, similar to the putative TCF/LEF-binding motifs (CTTTGWW) between –274 and −268, and between –54 and −48 of TSS, respectively. The reporter plasmids were transiently transfected with or without β-catenin siRNA in HCT116 cells, and luciferase activities were measured. As a result, we found that the luciferase activity of the cells transfected with RNF43-5′-1 or RNF43-5′-2 was significantly higher (approximately 3.0 and 2.5-fold, respectively) than that with empty vector, and that the activity of RNF43-5′-3 was similar to the empty vector (Fig. 2B). However, the knockdown of β-catenin did not affect the reporter activity of RNF43-5′-1 or RNF43-5′-2 (Fig. 2C). These data suggest that the 5.1-kb region contains the promoter but it does not include regulatory element(s) associated with Wnt/β-catenin signaling.

### Identification of WREs in *RNF43* Intron2

We next searched for putative regulatory regions in *RNF43* in public databases. A search in the ChIP-seq data of the ENCODE project (http://www.genome.ucsc.edu; The University of California Santa Cruz Genome Browser Database), identified four TCF4-enriched regions in the *RNF43* gene; one between −517 and +100 of TSS, two in intron2, and one in intron3. The two regions in intron2, but not the one in intron3, overlapped with RNA pol II-enriched regions. It is of note that these three regions were enriched with histone H3K4 mono-methylation. On the other hand, the region between –517 and −100 of TSS was enriched with pol II, histone H3K4 tri-methylation, and histone H3K27 acetylation, but not with histone H3K4 mono-methylation. Therefore we focused on the two regions in intron2 and tested whether they encompass TCF4-mediated transcriptional enhancer(s).

**Figure 3:**
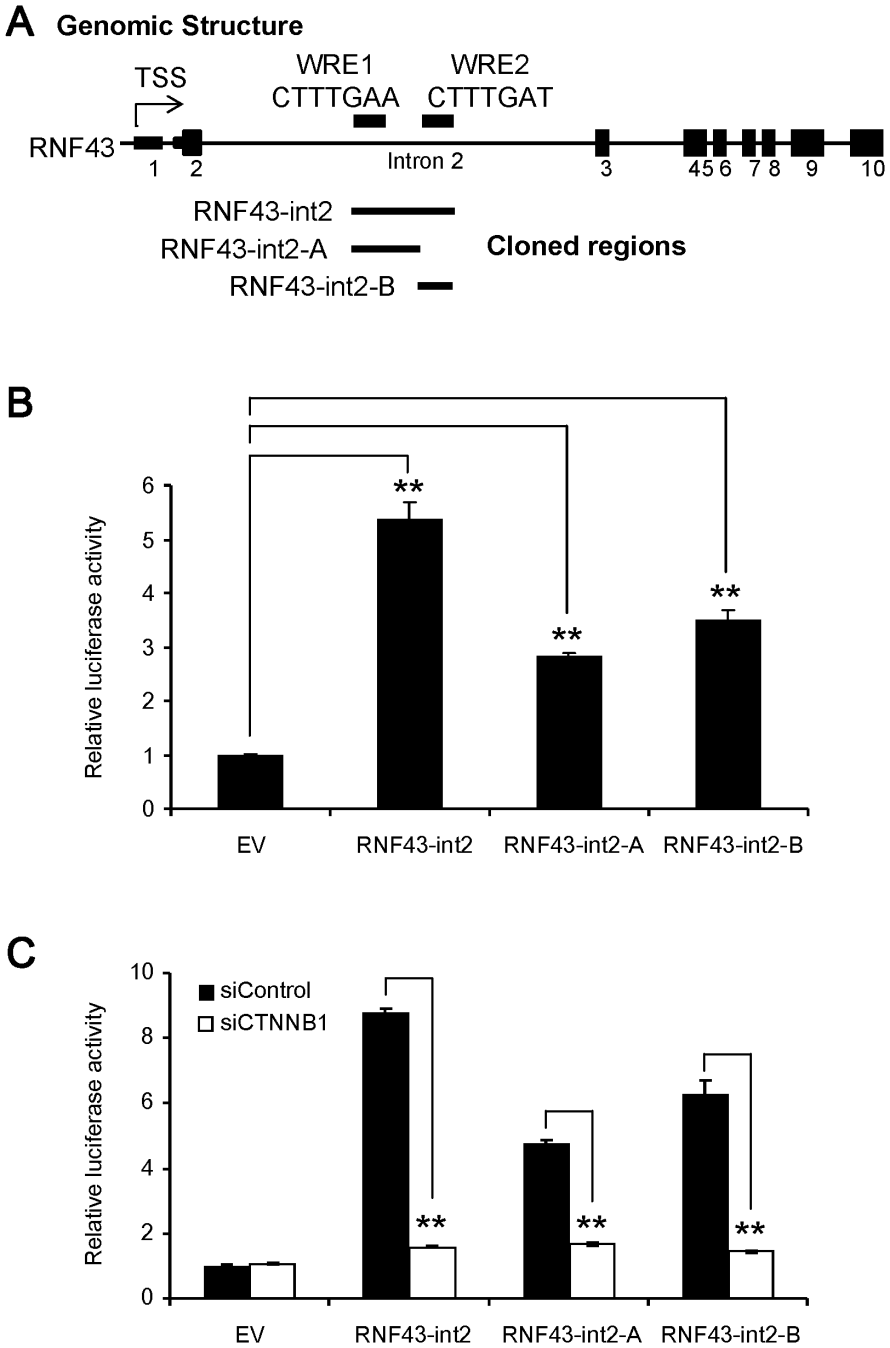
Luciferase assays with different regions in intron2. A) Schematic representation of Tcf4-enriched regions in the ENCODE data, and inserted regions in the promoter vector (pGL3-promoter). WRE: Wnt-responsive element. B) Enhancer activities were measured in triplicate using plasmids (RNF43-int2, RNF43-int2-A, and RNF43-int2-B) containing different regions in intron2 in HCT116 cells (mean ± standard deviation, **; P<0.01, Dunnett’s test). C) Effect of β-catenin depletion on the enhancer activities (**; P<0.01, Student’s t-test).

We then carried out a reporter assay using plasmids (RNF43-int2) containing a genomic region of 4.2 kb (chr17∶56468435–56472609, GRCh37) encompassing the two TCF4-enriched regions in intron2. As we expected, the plasmids showed approximately 5-fold higher activity than empty promoter vector in HCT116 cells (Figure 3B). Importantly, the activity was significantly decreased by the treatment with β-catenin siRNA (Figure 3C). The β-catenin-dependent activation was also observed in SW480 cells (data not shown). These data strongly suggested that the 4.2 kb region might be involved in the Wnt-dependent transcriptional activation.

**Figure 4:**
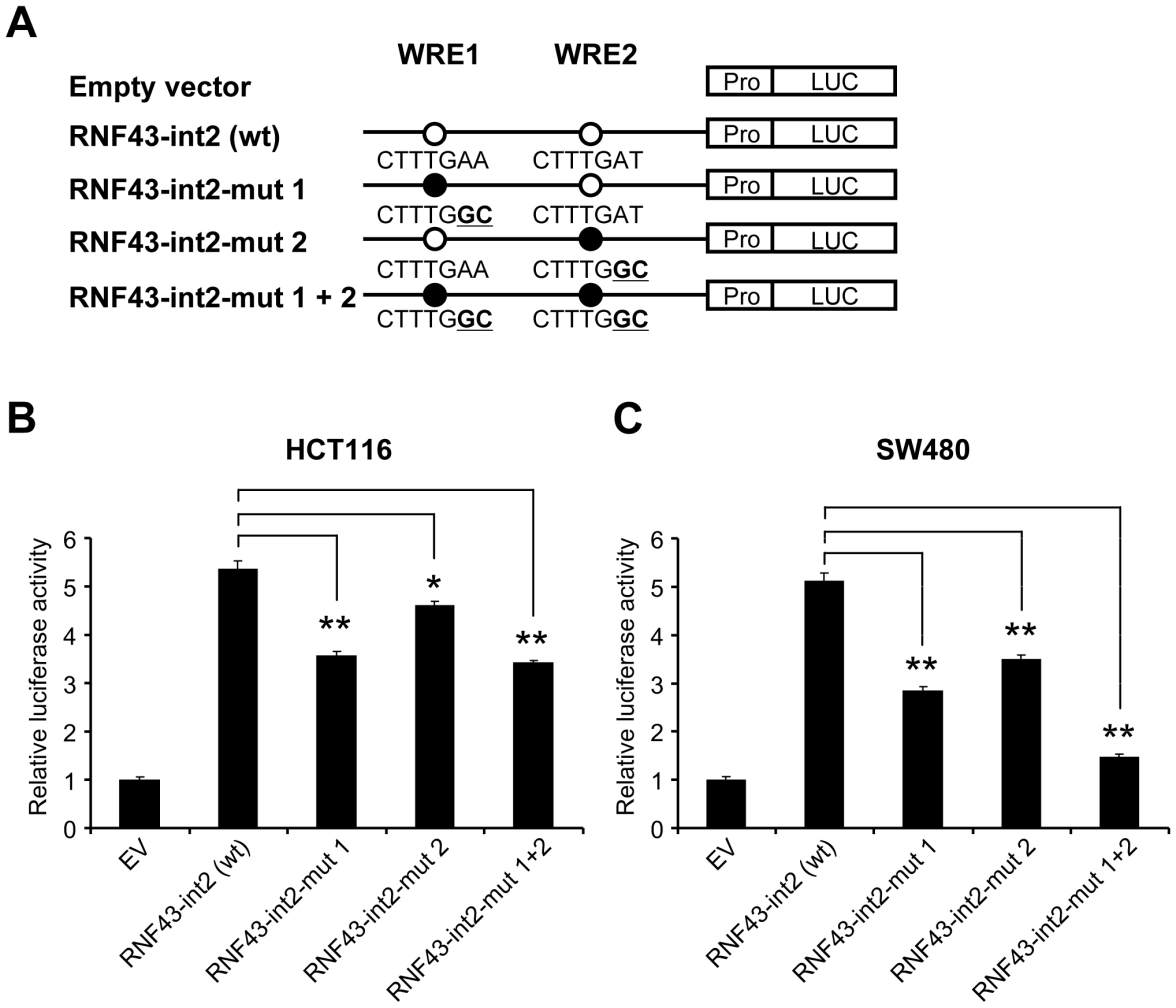
Involvement of WREs in the enhancer activity. A) Schematic representation of reporter plasmids containing substitution(s) in WRE1 and/or WRE2 (RNF43-int2-mut1, RNF43-int2-mut2, and RNF43-int2-mut1+2). B, C) Wild type or mutant reporter plasmids were transfected in HCT116 (B) and SW480 (C) cells, and luciferase activities were measured in triplicate (mean ± standard deviation, *; P<0.05, **; P<0.01, Dunnett’s test).

An additional search of transcription factor binding sites identified two putative TCF/LEF-binding motifs (CTTTGWW) in the regions; 5′-CTTTGAA-3′ and 5′-CTTTGAT-3′, which were termed putative Wnt-responsive element 1 and 2 (WRE1 and WRE2), respectively (Figure 3A). To clarify which element is crucial for the transactivation of *RNF43*, we prepared two forms of reporter plasmids containing either WRE1 or WRE2 (RNF43-int2-A and RNF43-int2-B, respectively). Although the luciferase activities of RNF43-int2-A and RNF43-int2-B were decreased at 57% and 65% of RNF43-int2, respectively, their activities were significantly higher than the control plasmids. In addition, co-transfection of β-catenin siRNA significantly suppressed both reporter activities (Figure 3C). We further generated mutant reporter plasmids containing substitution(s) in WRE1 and/or WRE2, from CTTTGWW to CTTTGGC (Figure 4A). Compared to the activity of wild type plasmids (RNF43-int2), reporter plasmids containing either substitution in WRE1 or WRE2 reduced the luciferase activity in HCT116 cells by 34% and 14%, respectively (Figure 4B). Similarly suppressed reporter activities were detected in SW480 cells (Figure 4C). Since combined substitutions in the WRE1 and WRE2 reduced the reporter activity by 36% in HCT116 cells and 71% in SW480 cells, other factor(s) may be involved in the enhanced reporter activity in HCT116 cells. Nevertheless, these data at least suggest that both elements should play a vital role in the β-catenin/TCF-dependent *RNF43* transactivation.

### Interaction of WRE1 and WRE2 with TCF4/β-catenin Complex

**Figure 5:**
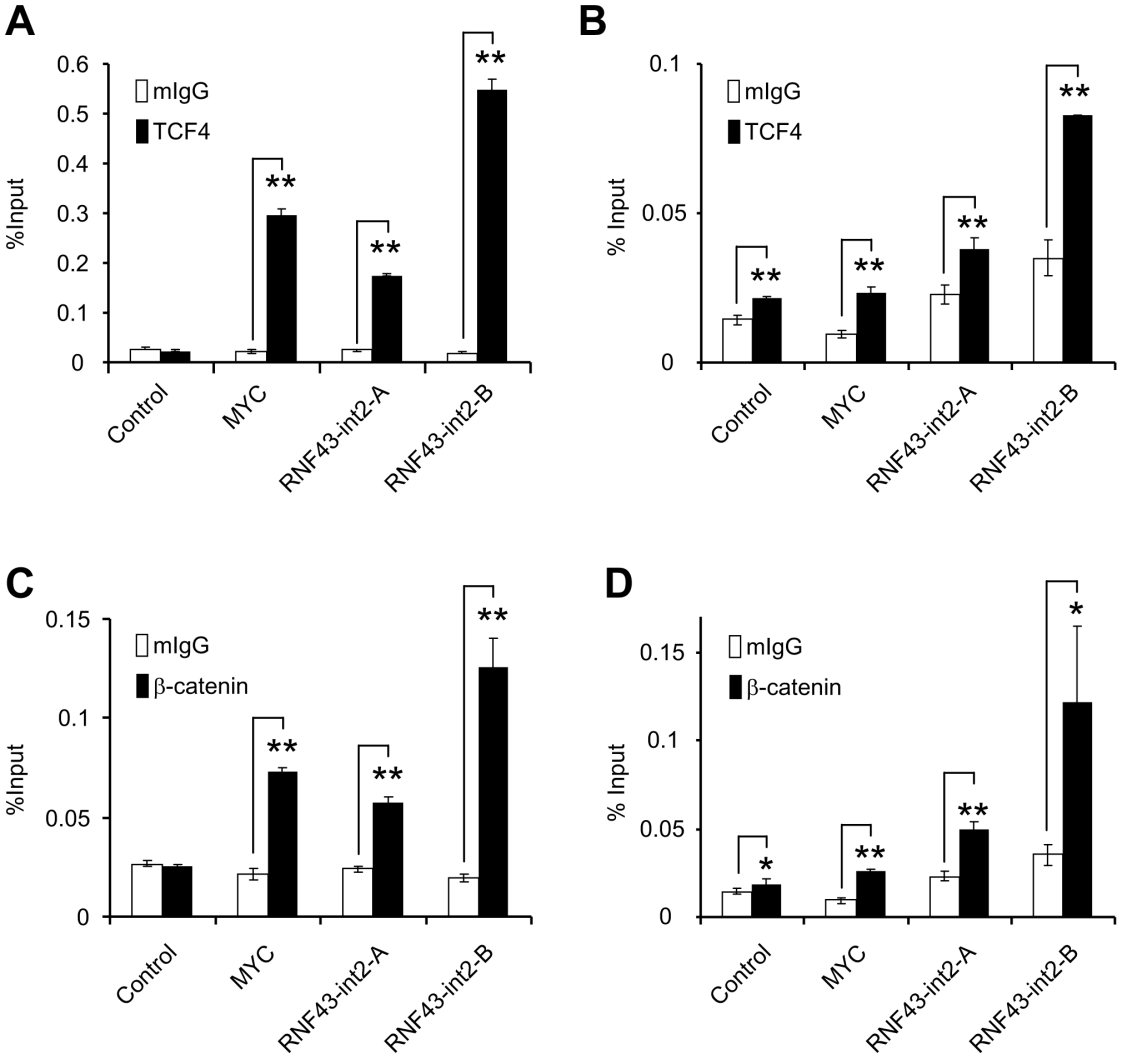
Association of the two WREs with Tcf4 and β-catenin. A, B) Quantification of precipitated regions by a ChIP assay with anti-TCF antibody was performed in HCT116 (A) and SW480 (B) cells using real-time PCR (mean ± standard deviation). Closed boxes indicate ChIP assay with anti-TCF4 antibody, and open boxes with control IgG. C, D) Quantification of precipitated regions by a ChIP assay with anti-β-catenin antibody in HCT116 (C) and SW480 (D) cells. A significant difference was determined by Student’s t-test (*; P<0.05, **; P<0.01).

To confirm whether TCF4 and β-catenin associates with WRE1 and WRE2, we conducted ChIP assays with TCF4-specific or β-catenin-specific antibody in HCT116 cells. Immunoprecipitation and subsequent quantitative PCR analysis revealed that the regions containing WRE1 and WRE2 were enriched by 7.3-fold and 28.1-fold with TCF4-specific antibody, respectively (Figure 5A). Of note, a WRE in the promoter of c-Myc, a direct target of β-catenin/TCF4 complex, was augmented about 13.6-fold in our analysis. Consistently, concordant enrichment of WRE1 and WRE2 was observed with β-catenin-specific antibody (Figure 5B). Similar results were obtained in SW480 cells, although the degree of enrichment was smaller than HCT116 (Figure 5C and 5D). These data indicated that the β-catenin/TCF4 complex interacts with WRE1 and WRE2 in intron2 of *RNF43*.

## Discussion

In this study, we have identified regulatory regions of RNF43 transcription and showed that *RNF43* is a direct target gene of Tcf4/β-catenin complex. Our initial challenge to identify regulatory region(s) using reporter assay containing the 5′ flanking region successfully showed that the region is involved in its transcriptional activation, but failed to find region(s) associated with Wnt/β-catenin signaling. Subsequent search of the ENCODE database helped us to explore candidate regions that may interact with Tcf4. Regarding the decrease of reporter activity with RNF43-5′-3 plasmids containing the 5′ flanking region, intron 1, and a part of intron 2, the 3′ region of intron 1 and/or the 5′ region of intron 2 may have a repressive element(s) for the transcription.

Genome-wide approaches including ChIP-on-Chip and ChIP-seq analyses have helped to illustrate a global association map of transcription factors, chromatin occupancy, and histone modifications. As for the Tcf4-interacting regions, Hatzis *et al.* found a total of 6,868 enriched regions using tiling array [36]. By means of luciferase-reporter assays, they examined 22 regions of approximately 1000 bp containing at least one of the enriched regions. Consequently, 10 of the 22 increased the activity, and 9 of them were down-regulated by the cotransfection with dominant negative Tcf4, suggesting that the enriched regions do not always play a role in transcriptional activation or associate with Tcf4/β-catenin. Their data included a Tcf4-enriched peak in intron2 of *RNF43* (chr17∶53823246, NCBI35/hg17), this peak was close to WRE2 (chr17∶53824064–53824070, NCBI35/hg17) suggesting a consistency of transcription factor-binding regions detected by ChIP-on chip in spite of different cell lines they used. They also reported that the Tcf4-binding sites are distributed along the genome, and 18% of them were located in intragenic regions further than 10 kb from the TSS. Consistently, the two WREs of *RNF43* were located at approximately 22 kb downstream of TSS. In addition, their data unveiled that 31% of peaks were located in the 5′-flanking region at a great distance from TSSs. In the case of c-Myc, WREs localized to a region over 400 kb upstream from the gene are involved in chromatin looping in response to the activation with serum mitogens [37]. Therefore additional WREs may play a role for the transcriptional regulation of RNF43 in combination with WRE1 and WRE2.

In addition to Tcf4, genome-wide approaches including serial analysis of chromatin occupancy (SACO) and ChIP-seq have been applied to identify the regions interacting with β-catenin [38], [39]. In agreement with our findings, the list of 412 β-catenin-interacting regions detected by SACO included a region containing the Tcf consensus motif in RNF43 (Chr17∶53824041 NCBI35/hg17), which corresponded to WRE2 in our data. The same group later carried out a ChIP-seq analysis and identified a total of 2,168 enriched regions with β-catenin in HCT116 cells. In the list of the 2168 regions, three were located in intron2 of RNF43 (chr17∶53819951–53820630, chr17∶53823671–53824270, and chr17∶53826701–53827401, NCBI36/hg18) [39]. Notably, WRE1 and WRE2 are within two of three enriched regions, suggesting that ChIP-seq is a powerful tool to discover binding regions of transcription factors.

In this study, we incorporated the data of histone modifications and occupancy of RNA pol-II. Consistent with the view that histone H3K4 mono-methylation and an interaction with RNA polymerase II are the hallmarks of transcriptional enhancers, the two WREs in intron2 played a vital role as a transcriptional enhancer in our luciferase assay. Meanwhile, the 5′-flanking region of 2.4 kb was associated with RNA polymerase II and histone H3K4 tri-methylation suggesting that this region served for the constitutive transcriptional activation through the remodeling of heterochromatin complex to euchromatin state. Although the ENCODE data depicted a peak of Tcf4-binding in the 5′-flanking region, enrichment of multiple transcription factors was observed in the same region. Therefore the peak of Tcf4 may be a false positive. Alternatively Tcf4 may interact with that region without the recruitment of β-catenin. Since the data of histone modifications and RNA pol-II occupancy are useful information to predict the chromatin structure of interacting regions and their transcriptional activity, future studies on a global association map of β-catenin and Tcf4 with histone modifications will gain an insight into the dynamic transcriptional regulation played by Tcf4/β-catenin and chromatin modification enzymes such as Brg1, TRRAP, TIP60, CBP/p300, and MLL [10].

Recently, it was reported that RNF43 and ZNRF3, transmembrane E3 ubiquitin ligases, selectively ubiquitinate frizzled receptors and targets them for degradation. RNF43 and ZNRF3, a homologue of RNF43, are highly conserved in vertebrates, and associate in the membrane with frizzled receptors and low density lipoprotein receptor-related proteins, LRP5/6. RNF43 promotes endocytosis of frizzled receptors including FZD1 and FZD3, and suppresses Wnt/β-catenin responses [26]. ZNRF3 also promotes the turnover of frizzled receptors and LRP6. Interestingly inhibition of ZNRF3 enhances Wnt/β-catenin signaling and suppresses Wnt/planar cell polarity signaling [25]. These data suggested that ZNRF3 and RNF43 regulate canonical and non-canonical Wnt pathway. Our data, in line with others, suggest that RNF43 functions as a negative feedback regulator modulated by Tcf4/β-catenin complex. This notion is reminiscent of *AXIN2*, and *DKK1*, which are also downstream targets of the canonical Wnt signaling pathway and negatively regulate the signals in different manners [40], [41], [42], [43], [44]. Regarding the tumorigenesis of pancreatic and ovarian cancers, inactivating mutations in *RNF43* are supposed to abrogate Wnt signaling including canonical and non-canonical pathways. However, the effect of negative feedback by the enhanced expression of RNF43 has not been clarified in colorectal or liver cancer. Although the augmented transcriptional activity of Tcf4/β-catenin complex by inactivation mutations in *APC* or *AXIN2*, or activating mutations in *CTNNB1* may not be affected by the suppression of frizzled receptors, complex network of signaling pathways may render undetermined characteristics to colorectal and liver cancer cells, as restoring expression of *SFRP4* and *DKK1* in colorectal cancer cells attenuates Wnt signaling [44], [45]. Further investigations on the effect of enhanced or suppressed RNF43 function may shed light on the undetermined networks associated with canonical and non-canonical Wnt pathways, and may contribute to the development of diagnostic, therapeutic, and/or preventive strategies to human diseases.
